# 4D flow MRI of the aorta becomes practical: performance and observer variability for a new semi-automated workflow for 3D visualization and quantification of aortic hemodynamics

**DOI:** 10.1186/1532-429X-15-S1-M2

**Published:** 2013-01-30

**Authors:** Susanne Schnell, Pegah Entezari, Riti J Mahadevia, Daniel Rinewalt, Jacob Fluckiger, Jeremy Collins, James Carr, Bernd A Jung, Michael Markl

**Affiliations:** 1 Dept. of Radiology, Northwestern University, Feinberg Medical School, Chicago, IL, USA; 2 Cardiac Surgery, Rush University, Chicago, IL, USA; 3 Radiology, Medical Physics, University Medical Center Freiburg, Freiburg, Germany; 4 Biomedical Engineering, Northwestern University, Evanston, IL, USA

## Background

To systematically investigate the performance and inter-observer variability of a new standardized workflow for the analysis of aortic hemodynamics based on 4D-flow MRI in a study with 30 subjects. The semi-automated workflow was developed to ensure faster and standardized data analysis including systematic placement of analysis planes, 3D-flow visualization, and quantification of standard clinical flow parameters.

## Methods

ECG and respiration-synchronized flow-sensitive 4D MRI data (spatio-temporal resolution=2.1-2.9x2.1-2.5x2.5-3.2mm3 / 37.6-40ms) were acquired in 10 healthy volunteers, 10 patients with dilation of the ascending aorta and 10 patients with bicuspid valves (BAV). After correction for noise, eddy-currents and aliasing (Bock et al, ISMRM 2007), the data were loaded into a 3D visualization software (Ensight, CEI, USA). A set of macros was developed (in python) to minimize user interaction, permit a reproducible definition of analysis planes, and reduce time needed for comprehensive 4D-flow analysis. This included 1) automated calculation of 3D PC-MRA; 2) creation of 9 standardized analysis planes to be positioned manually along the aorta; 3) quantification of net, forward and reverse flow, regurgitation and peak systolic velocity; 4) 3D-flow visualization by time-resolved pathlines; 5) saving and export as images and movies (for presentation purposes and transfer to the hospitals PACS system). Steps 3-5 were fully automated. For evaluation, the data was analyzed by two independent observers, and agreement was tested in 3 analysis planes (mid-ascending aorta, mid-arch and proximal descending aorta).

## Results

**Figure 1:**
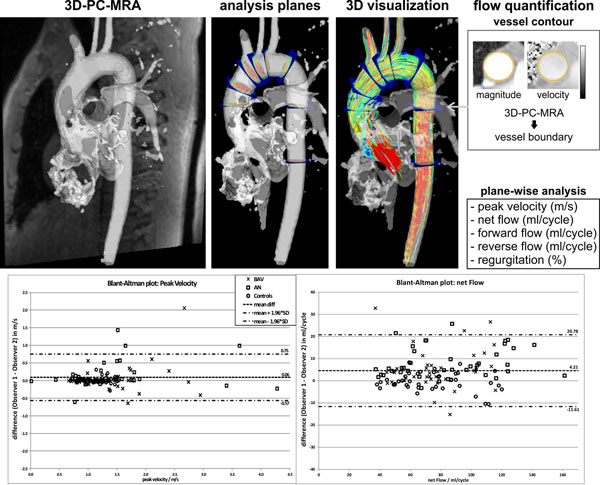
The upper row of the figure illustrates the steps 1 - 4 of the new standardized semi-automated workflow including the automated calculation of the 3D PC-MRA, the positioning of the 9 analysis planes equally distributed along the aorta, the flow visualization by calculating time-resolved pathlines and the flow quantification by determining peak velocity, net, forward,and reverse flow as well as regurgitation. The second row shows the Blant-Altman plots of peak velocity and net flow for the two observers summarized for 3 analysis planes.

In all 30 subjects the analysis was successfully completed and provided comprehensive information on aortic hemodynamics including planar flow quantification, and 3D-flow visualization with a 3D viewer (Enlighten, CEI, USA) as well as in standardized views as standalone videos and images. As summarized in figure 1 and table 1, inter-observer agreement was excellent for peak velocity, reverse flow and regurgitation (mean differences 4.59, 0.94 and 3.36% of the average parameters). The net and forward flow demonstrated moderate agreement (mean differences 5.4 and 5.1%). The mean analysis time for the complete analysis was 80min ±28. Note that the time was prolonged by the processing time needed generate the image and video files. For steps 1-4 only the analysis time was substantially reduced to 37.2min ± 12.5. The average time for noise, eddy currents and aliasing correction was 13.4min ± 5.8.

**Table 1:** Quantification results showing the mean values, mean difference between the two observers and its limits of agreement (LOA)

	Mean Controls	Mean BAVs	Mean Aneurysms	Mean Total	Mean Difference Controls	Mean Difference BAVs	Mean Difference Aneurysms	Mean Difference Total	LOA (+/-1.96*SD)
Peak Velocity (m/s)	1.16	1.31	1.22	1.32	0.02	0.10	0.16	0.09	0.66
Net flow (ml/cycle)	69.87	71.13	74.53	77.45	-0.86	6.40	8.22	4.59	16.19
Forward flow (ml/cycle)	70.53	73.73	77.16	79.40	-1.21	6.13	8.31	4.41	16.00
Reverse flow (ml/cycle)	-0.85	-2.48	-2.72	-2.00	-0.01	0.03	-0.26	-0.11	-3.11
Regurgitation (%)	1.23	3.54	3.93	2.71	0.05	0.32	0.15	-0.04	3.56

## Conclusions

Standardized 4D-flow MRI data analysis based on a macro oriented workflow can be performed with good inter-observer agreement and provides comprehensive information of aortic hemodynamics. Improved computation speed and better algorithms for the calculation of 3D visualization are needed to further reduce analysis time to clinically acceptable times.

## Funding

DFG (German Research Foundation) SCHN 1170/2-1

